# Bir1 Deletion Causes Malfunction of the Spindle Assembly Checkpoint and Apoptosis in Yeast

**DOI:** 10.3389/fonc.2012.00093

**Published:** 2012-08-09

**Authors:** Qun Ren, Liang-Chun Liou, Qiuqiang Gao, Xiaoming Bao, Zhaojie Zhang

**Affiliations:** ^1^Department of Zoology and Physiology, University of WyomingLaramie, WY, USA; ^2^State Key Laboratory of Microbial Technology, Shandong UniversityJinan, China

**Keywords:** apoptosis, BIR1, cell cycle, oxidative stress, spindle assembly checkpoint

## Abstract

Cell division in yeast is a highly regulated and well studied event. Various checkpoints are placed throughout the cell cycle to ensure faithful segregation of sister chromatids. Unexpected events, such as DNA damage or oxidative stress, cause the activation of checkpoint(s) and cell cycle arrest. Malfunction of the checkpoints may induce cell death. We previously showed that under oxidative stress, the budding yeast cohesin Mcd1, a homolog of human Rad21, was cleaved by the caspase-like protease Esp1. The cleaved Mcd1 C-terminal fragment was then translocated to mitochondria, causing apoptotic cell death. In the present study, we demonstrated that Bir1 plays an important role in spindle assembly checkpoint and cell death. Similar to H_2_O_2_ treatment, deletion of BIR1 using a BIR1-degron strain caused degradation of the securin Pds1, which binds and inactivates Esp1 until metaphase-anaphase transition in a normal cell cycle. BIR1 deletion caused an increase level of ROS and mis-location of Bub1, a major protein for spindle assembly checkpoint. In wild type, Bub1 was located at the kinetochores, but was primarily in the cytoplasm in bir1 deletion strain. When BIR1 was deleted, addition of nocodazole was unable to retain the Bub1 localization on kinetochores, further suggesting that Bir1 is required to activate and maintain the spindle assembly checkpoint. Our study suggests that the BIR1 function in cell cycle regulation works in concert with its anti-apoptosis function.

## Introduction

Similar to many other organisms, cell cycle in the yeast *Saccharomyces cerevisiae* is highly regulated. Various checkpoints are in place throughout the cell cycle to ensure faithful segregation of sister chromatids. Unexpected events, such as DNA damage or oxidative stress, cause the activation of checkpoint(s) and cell cycle arrest (Lew et al., 1997). Malfunction of the checkpoints may induce cell death. UV-radiation, for instance, causes alteration of cell cycle and apoptosis in yeast (Del Carratore et al., 2002). Mis-regulation of cell cycle is also found in apoptotic cell death of neuronal cells. Under normal conditions, neuronal cells are maintained in a quiescent G0 state. In the ischemia/reperfusion stroke model of both mice and rats, cerebral neurons were observed to re-enter the cell cycle prior to cell death (Osuga et al., 2000; Katchanov et al., 2001).

Cell cycle regulation under oxidative stress is well studied in both mammalian and yeast cells. Studies show that oxidative stress causes cell cycle arrest, due to the reactive oxygen species (ROS) induced DNA damage (Migliore and Coppede, 2002; Shapira et al., 2004). ROS also induces apoptosis in yeast (Ghibelli et al., 1995; Madeo et al., 1999). It is less clear under what conditions the ROS-exposed cells undergo cell cycle arrest, or apoptosis. One report shows that the H_2_O_2_ treated human fibroblasts undergo either cell cycle arrest or apoptosis. It depends, at least in part, on where the cell resides in the cell cycle. The majority of the apoptotic fibroblasts were in S phase, whereas growth-arrested cells were predominantly in G1 or G2/M phase (Chen et al., 2000). Another factor that may affect the fate of the cells is the level of the oxidative stress. In yeast, for example, low dose of H_2_O_2_ induces cell cycle arrest, while high dose causes cell death (Madeo et al., 2004; Shapira et al., 2004).

Failure of the cell cycle checkpoint activation may play an important role in stress-induced cell death (Sczaniecka and Hardwick, 2008). We previously reported that under oxidative stress, the yeast cohesin protein Mcd1, a human Rad21, was cleaved by the caspase-like protease Esp1. The cleaved Mcd1 C-terminal fragment was then translocated to mitochondria, causing apoptotic cell death (Yang et al., 2008). The Esp1 is a cell cycle regulated protein. It is activated by the anaphase promoting complex (APC), which degrades the anaphase inhibitor Pds1. The cleavage of Mcd1 under oxidative stress suggests the interruption or mis-regulation of the spindle assembly checkpoint, which regulates the APC activation.

Bir1 is a chromosomal passenger protein involved in coordinating cell cycle events for proper chromosome segregation (Widlund et al., 2006). It has been shown that Bir1 is required for recruiting condensin. Deletion of BIR1 in *Schizosaccharomyces pombe* inactivates the spindle assembly checkpoint and causes the destruction of securin in the absence of normal anaphase (Morishita et al., 2001). Bir1 also exhibits anti-apoptotic activity. Bir1 deletion in yeast causes apoptotic cell death and is hypersensitive to oxidative stress (Walter et al., 2006). It is not clear whether the anti-apoptosis function of yeast Bir1 is related to its function in cell cycle regulation (Yang et al., 2008), or if yeast Bir1 functions as an inhibitor of apoptosis, similar to the caspase inhibitors found in mammalian cells (Silke and Vaux, 2001).

In the present study, we demonstrated that Bir1 plays an important role in spindle assembly checkpoint and cell death. We showed that deletion of BIR1 caused elevated level of ROS and apoptotic cell death. We further demonstrated that the apoptosis was likely induced by the mis-regulation of spindle assembly checkpoint, as indicated by the degradation of the anaphase inhibitor Pds1 and the mis-localization of the spindle assembly checkpoint protein Bub1.

## Materials and Methods

### Yeast strains and culture conditions

Yeast strains were derivatives of the BY4742 (MATα his3Δ1 leu2Δ0 lys2Δ0 ura3Δ). The plasmids for making heat-inducible BIR1-degron were purchased from EUROSCARF^1^. The BIR1-degron strain was constructed as described previously (Sanchez-Diaz et al., 2004). The C-terminal BUB1-HA tag was constructed as described before (Janke et al., 2004).

Cells were grown at 30°C in YPD (1% yeast extract, 2% peptone, and 2% glucose), except where noted in the text. For BIR1-degron induction, cells were first grown YPDCu (YPD with 0.1 mM CuSO4) at 24°C to a concentration of 1 × 10^8^ cells/ml. Cells were then diluted into YPRCu medium (1% yeast extract, 2% peptone, 2% raffinose, and 0.1 mM CuSO4) and continued to grow at 24°C to 1 × 10^7^ cells/ml. Cells were then transferred to YPG medium pre-warmed to 37°C to induce the BIR1-degron.

### Semi-quantitative RT-PCR

Total RNA from yeast was extracted using RNeasy Protect Mini Kit (QIAGEN, CA, USA). The reverse transcript (RT)-PCR and the amplification procedure were performed as described previously (Gao et al., 2009). Yeast actin gene (*ACT1*) was used as control.

### Western blot analysis

Cells were collected by centrifugation and lysed in lysis buffer containing 20 mM Tris (pH 7.4), 150 mM NaCl, 1 mM EDTA, 1 mM EGTA, 1% Triton, 0.1% SDS, and protease inhibitor cocktail. Two-hundred micrometers of glass beads were added and vortexed vigorously. Sample was boiled, spun down and the supernatant was used to run the SDS-gel. Lysates were separated on 10% SDS-polyacrylamide gels and transferred to polyvinylidene difluoride membranes. The membranes were then blocked in 4% non-fat milk and then incubated with anti-HA antibody (clone 3F10, Roche Diagnostics, IN, USA).

### Confocal microscopy

Nuclear spread preparation of BUB1-HA was according to Zhang et al. (2005). The primary and secondary antibodies used for immunostaining were rat anti-HA (Roche Diagnostics, IN, USA) and Alexa Fluor 488 conjugated goat anti-rat IgG (Molecular Probes), respectively. Nucleus was counterstained with 4,6-diamidino-2-phenylindole (DAPI; 0.5 mg/ml). Images were taken using a Zeiss 710 Laser Scanning Confocal Microscope (Jena, Germany).

### Transmission electron microscopy

Transmission electron microscopy (TEM) sample was prepared according to Wright (2000). Briefly, cells cultured in either the non-inducing medium (YPDCu at 24°C) or the inducing medium (YPG at 37°C) and then were harvested by gentle centrifugation, washed in phosphate buffered saline (PBS; pH = 7.2), resuspended in 2.5% (v/v) glutaraldehyde in PBS and fixed 40 min at room temperature. Cells were further fixed by 2% potassium permanganate in water for 1 h at room temperature. Fixed cells were dehydrated with 30, 50, 75, 85, 95, and 100% ethanol. Cells were transitioned with propylene oxide, infiltrated in Spurr resin (Electron Microscopy Sciences, PA, USA) Resin was polymerized at 65°C overnight. Sixty nanometers of ultrathin sections were cut with a diamond knife, stained with 2% uranyl acetate and lead citrate, examined using a Hitachi H-7000 electron microscope, and equipped with a high resolution (4 K × 4 K) cooled CCD digital camera (Gatan, Inc.).

### Detection of reactive oxygen species

The detection of ROS was performed according to Ren et al. (2005). Briefly, cells were grown at either the non-inducing or inducing media to early log phase. Cells were then washed with PBS for three times and stained for 10 min with dihydroethidium (5 μM; Sigma), viewed with a Zeiss 710 laser scanning confocal microscope with excitation at 514 nm. The fluorescence intensity of about 300 cells from three different experiments was measured using ImageJ software.^2^

### Statistic analysis

Data were presented as the mean ± SD from three independent experiments. Statistical significance was determined by Student’s *t*-test. *P* < 0.05 was considered to be statistically significant.

## Results

### BIR1-degron causes degradation of both BIR1 mRNA and protein

**Figure 1:**
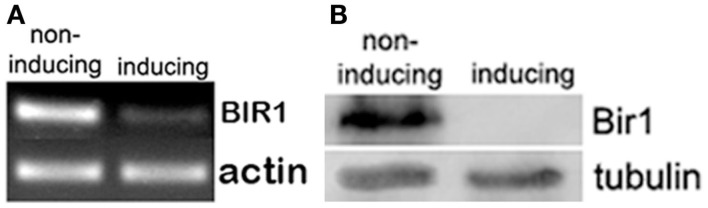
**BIR1-degron causes degradation of both BIR1 mRNA (A) and protein (B)**. BIR1-degron strain was grown in the non-inducing condition (YPDCu, 24°C) to early log phase. Cells were then transferred to inducing condition (YPG, 37°C) for 1°h. PT-PCR **(A)** or Western blot **(B)** was used to examine the mRNA or protein level.

To further understand the role of Bir1 in cell cycle regulation and cell death, we constructed a heat-inducible BIR1-degron strain (Sanchez-Diaz et al., 2004). RT-PCR and Western blot were used to check the changes of BIR1 mRNA and protein levels. When cells were shifted from normal growing condition (YPDCu, 24°C) to inducing condition (YPG, 37°C) for 1 h, RT-PCR showed the expression level of Bir1 mRNA was dramatically decreased. Western blot using anti-HA, showed that Bir1 protein was completely depleted (Figure 1).

### BIR1 deletion causes defect on spindle assembly checkpoint

**Figure 2:**
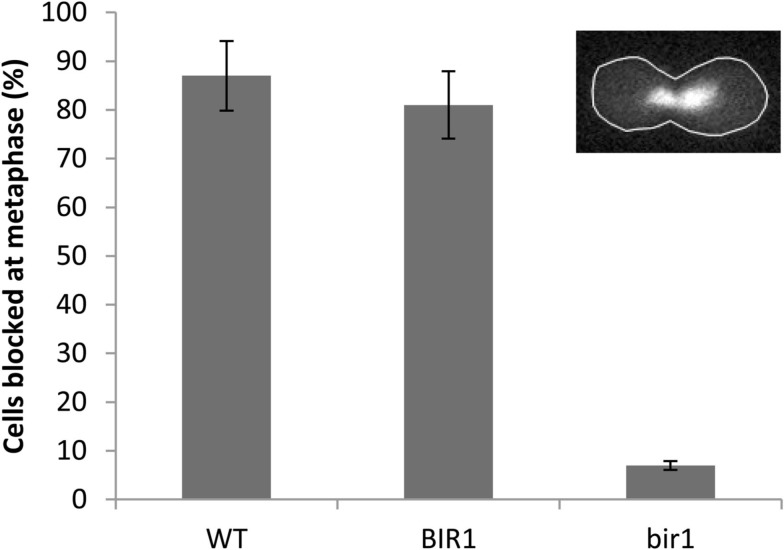
**Bir1 deletion causes spindle checkpoint defects**. Wild type was grown in YPD at 30°C (WT), or BIR1-degron strain was grown in either non-inducing condition (YPDCu, 24°C; BIR1), or in inducing condition (YPG, 37°C; bir1) to log phase. 10 μg/ml of nocodazole was added to culture media for 2 h. Cells were stained with DAPI and examined using a fluorescence microscope. Cells were considered as metaphase when (1). A cell had a daughter cell similar in size to the mother cell; and (2). The nuclei were in the middle of the dividing cells (inset). About 300 cells were counted from two independent experiments.

Next, we asked if deletion of BIR1 via the BIR-degron affects the function of spindle assembly checkpoint. When nocodazole (10 μg/ml), which blocks the polymerization of microtubules, was added to the culture medium of wild type cells for 2 h, about 90% of the cells were blocked at the metaphase, which was judged by (1). A cell had a daughter cell similar to the size of the mother cell; and (2). The nuclei were in the middle of the dividing cells (Figure 2, inset). A similar result was obtained with BIR1-degron strain growing at the non-inducing condition (YPDCu, 24°C; data not shown). When growing at the inducing condition, however, less than 10% of the bir1-degron cells were blocked at metaphase (Figure 2), suggesting that Bir1 is required for activating the spindle assembly checkpoint.

### BIR1 deletion induces apoptotic cell death

**Figure 3:**
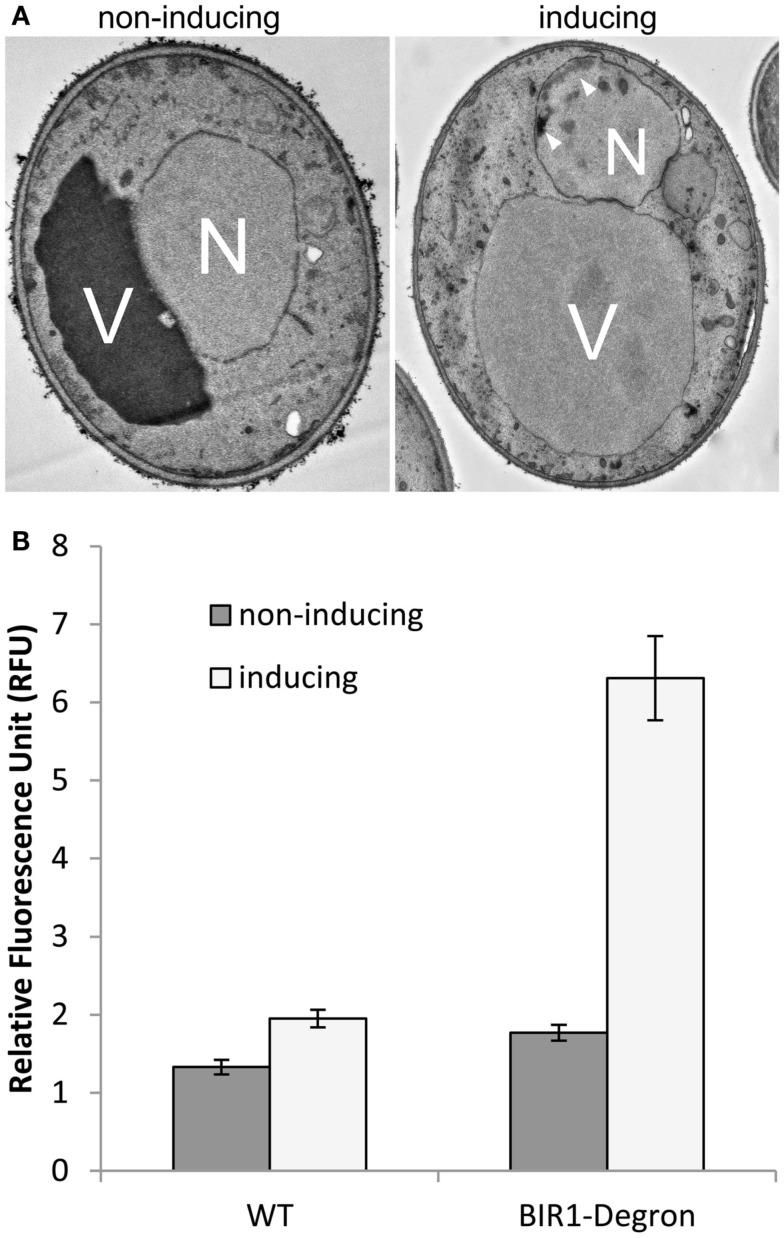
**Bir1 deletion causes apoptotic cell death. (A)**. TEM images showing bir1 deletion caused chromatin condensation (arrowheads) in the nucleus. V, vacuole; N, nucleus. **(B)**. Quantitative analysis of ROS production.

**Figure 4:**
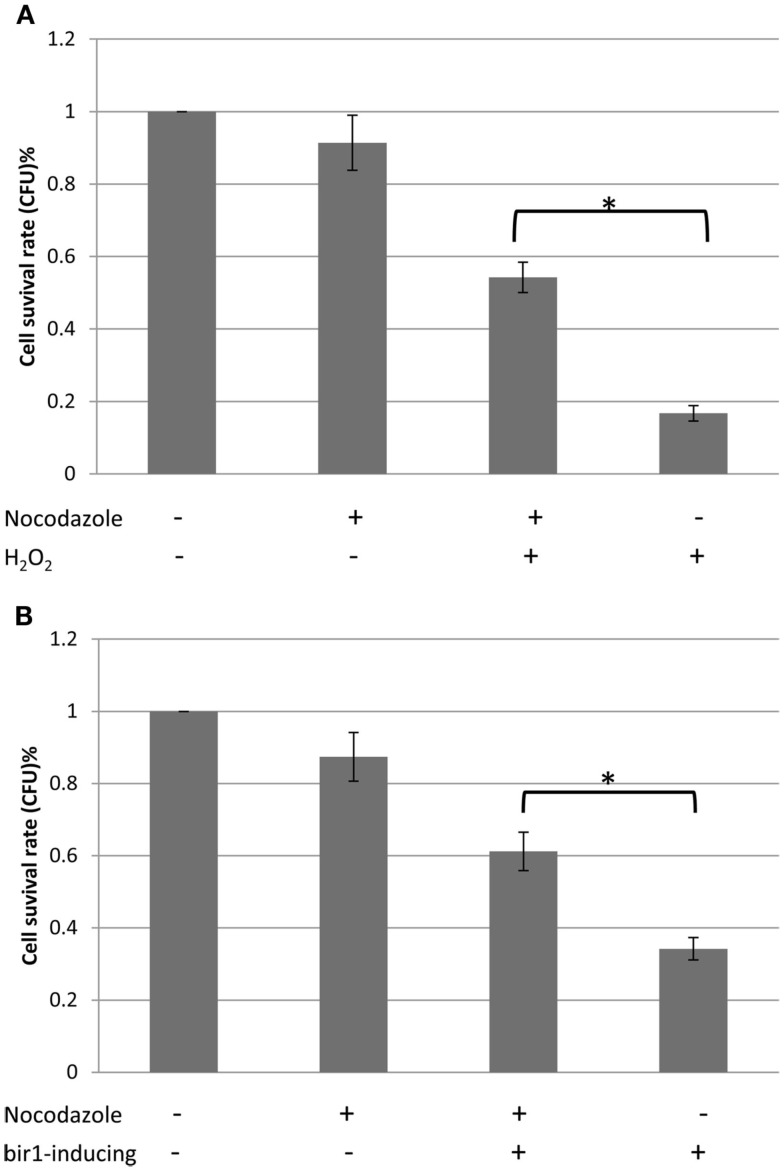
**Cell survival rates with or without nocodazole**. **(A)**. Wild type cells were grown to early log phase. 10 μg/ml nocodazole was then added to the culture medium for 2°h, to block cells to pro-metaphase. Four millimolars of H_2_O_2_ was added for 1°h and 500 cells were plated on YPD plate. Colony forming unit (CFU) was calculated against control. **(B)**. BIR1-degron cells were grown in YPDCu (24°C) to early phase. 10 μg/ml nocodazole was then added to the culture medium for 2°h, to block cells to pro-metaphase. Cells were then transferred to YPG (37°C) for 1°h, followed by plating assay. *P < 0.05.

Previous study has shown that deletion of BIR1 causes apoptotic cell death (Walter et al., 2006). Using TEM, we showed the chromatin condensation in the nucleus, when BIR1-degron is activated, further confirming the occurrence of apoptosis in the bir1-degron strain (Figure 3A). The level of ROS, another marker of the apoptotic cell death, was also much higher when BIR1-degron was growing in the inducing medium (Figure 3B). The slight increase of ROS in wild type was likely caused by the temperature change, from the non-inducing condition (24°C) to inducing condition (37°C). To see if the malfunction of spindle assembly checkpoint is involved in the cell death, we used nocodazole (10 μg/ml) to block the cells to pro-metaphase prior to the induction of BIR1-degron or added H_2_O_2_ (4 mM), which has shown to induce apoptotic cell death in yeast (Madeo et al., 1999). As shown in Figure 4, when cells were first blocked at pro-metaphase by nocodazole, the cell survival rate was significantly higher either in the presence of H_2_O_2_ (Figure 4A), or during the induction of BIR1-degron (Figure 4B), compared to cells without nocodazole treatment. These results further suggest that spindle assembly checkpoint plays an important role in bir1 deletion or oxidative stress-induced cell death.

### BIR1 deletion causes the degradation of the anaphase inhibitor Pds1

**Figure 5:**
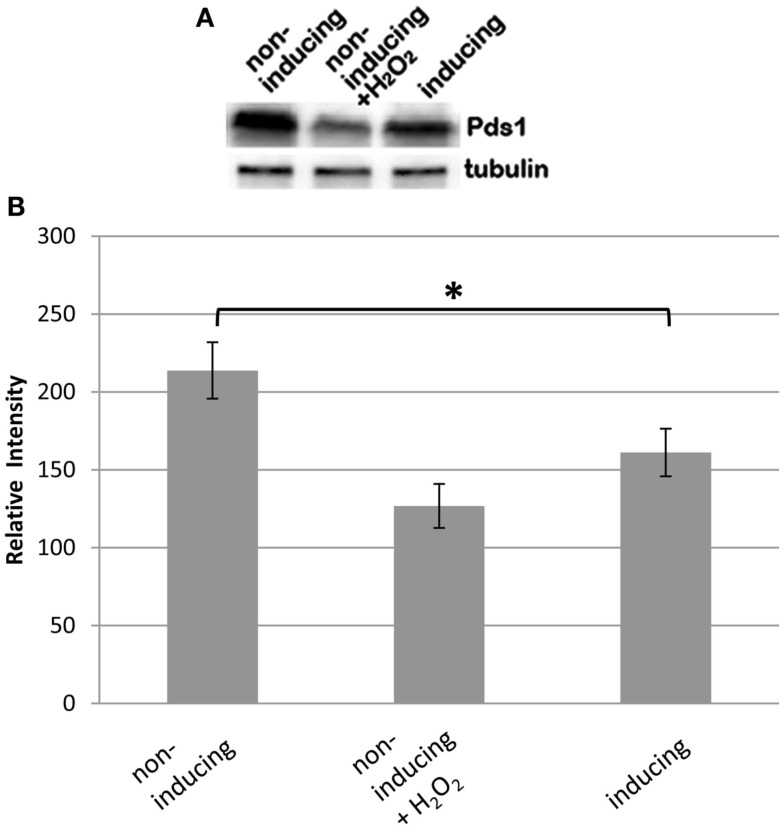
**Bir1 deletion causes the degradation of the anaphase inhibitor Pds1**. BIR1-degron cells were grown in YPDCu (24°C) to early phase. Four millimolars of H_2_O_2_ was added for 1°h (middle lane), or cells were transferred to YPG (37°C) for 1°h. **(A)** Western blot analysis of Pds1 degradation and **(B)** quantitative analysis of the Western blot (*n* = 3). β-Tubulin was used as control. *P < 0.05.

To further confirm the defect of spindle assembly checkpoint caused by BIR1 deletion, Western blot was used to examine the levels of Pds1, the anaphase inhibitor. In a normal cell cycle, Pds1 is degraded only during metaphase to anaphase transition, by APC. We showed previously that oxidative stress caused cleavage of the cohesin protein Mcd1 by the caspase-like protease Esp1 (Yang et al., 2008). Western blot showed that the addition of H_2_O_2_ caused the degradation of Pds1 (Figure 5A), confirming the activation of APC under oxidative stress. When BIR1 was deleted via BIR1-degron, Pds1 was also degraded (Figure 5A), further suggesting the disruption of the spindle assembly checkpoint. The Pds1 degradation caused by H_2_O_2_ is greater than bir1 deletion (Figure 5B), suggesting that oxidative stress has a more severe effect on cell cycle disruption.

### Bub1 fails to be localized on centromeres in the presence of H_2_O_2_ or bir1 deletion

**Figure 6:**
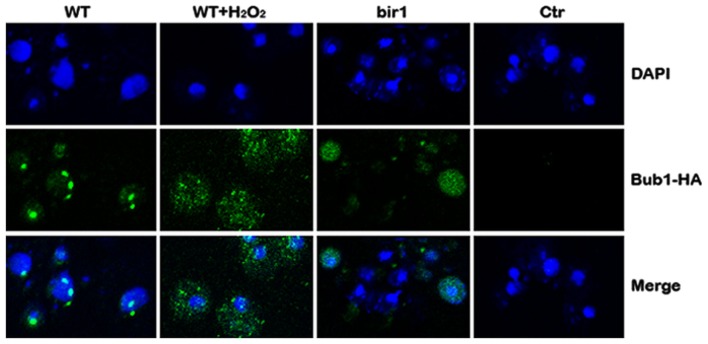
**Bub1 fails to be localized on centromeres in the presence of H_2_O_2_ or bir1 deletion**. Wild type (WT) cells were grown in YPD to log phase. Four millimolars of H_2_O_2_ was added to the medium for 1°h (WT + H_2_O_2_); or Bir1-degron strain was grown to log phase at the non-inducing condition (YPDCu, 24°C); Cells were then transferred to inducing medium (YPG, 37°C) for 1°h (bir1). Bub1 localization was assayed by immunostaining and confocal microscopy. Control (Ctr) was conducted by replacing primary antibody (anti-HA) with buffer.

Bub1 is a protein kinase that is involved in the spindle assembly checkpoint. Bub1 localizes at the centromeres throughout mitosis in fission yeast (Bernard et al., 1998) and mammalian cells (Howell et al., 2004). Using an HA-tagged BUB1 strain and immunocytochemistry, we showed that in log phase cells, Bub1 formed small foci inside the nucleus, similar to the pattern of centromere staining (Bernard et al., 1998). When cells were treated with H_2_O_2_, however, no Bub1 foci were observed inside the nucleus. Bub1 was diffused into cytoplasm. A similar phenotype was observed when bir1 was deleted by the bir1-degron (Figure 6). These results suggest that Bir1p is required for Bub1 localization on centromeres. Another possibility is that H_2_O_2_ or bir1 deletion induces apoptosis which causes centromeres to be disrupted therefore Bub1 cannot localize to centromeres.

## Discussion

Eukaryotic cells have evolved a complex network, known as cell cycle control system, to regulate the progression of cell cycle and cell division. One important component of the regulation network is the checkpoint control, which senses flaws in critical events, such as DNA replication and chromosome segregation (Lew et al., 1997). When checkpoints are activated, cell cycle is delayed, until damages are repaired or removed. Malfunction of checkpoint control could induce cell death, or cancer.

**Figure 7:**
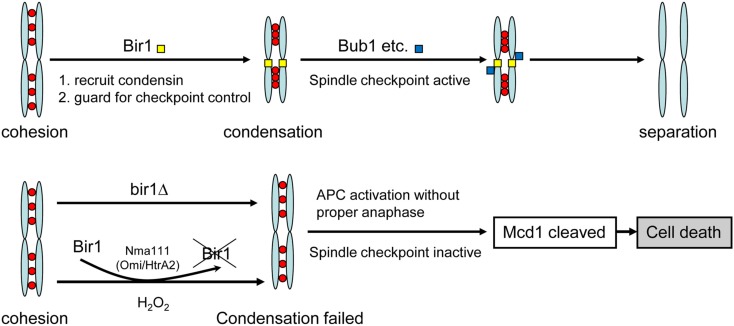
**Model of cell death induced by bir1 deletion or oxidative stress**. Under normal condition, Bir1 is localized at the centromeres as part of the spindle assembly checkpoint complex. In the lack of Bir1 (mutation or by removed by Nma111 under oxidative stress Walter et al., 2006), the spindle checkpoint is inactivated, causing improper APC activation and the consequent cell death.

Two kinds of BIR-containing proteins have been identified; one functions as inhibitor of apoptosis and the other is involved in cell cycle regulation. Similar to mammalian survivin (also known as Birc5), the budding yeast Bir1 contains a single RING-finger domain. It is cell cycle regulated and localizes to the centromeres until metaphase-anaphase transition but remains in the equatorial zone as the sister chromatids separate (Uren et al., 1998, 2000; Widlund et al., 2006). Anti-apoptotic function has also been reported for survivin and the yeast homolog Bir1. Du et al. (2000) revealed that survivin bound to Diablo/Smac, a mitochondrial protein that promotes apoptosis by activating caspases in the cytochrome c/Apaf-1/caspase-9 pathway. Madeo and his colleagues (Walter et al., 2006) showed that the yeast Bir1 is a substrate of Nma111, the homolog of the human pro-apoptotic serine protease Omi/HtrA2. Under oxidative stress, Bir1 was degraded by Nma111, causing apoptotic cell death. In this study, we further demonstrated that deletion of yeast BIR1 induced apoptotic cell death. However, the cell death induced by deletion of bir1 is not due to its anti-apoptosis function, but rather its function in cell cycle regulation. We demonstrated that Bir1 is required for the activation of the spindle assembly checkpoint. This notion is supported by (1). BIR1 deletion causes defect on spindle assembly checkpoint (Figure 2); and (2). The spindle checkpoint protein Bub1 fails to localize on centromeres when BIR1 is deleted (Figure 6). As a result, the anaphase inhibitor Pds1 is degraded in bir1 deletion in the absence of normal anaphase. The degradation of Pds1 causes the cleavage of the cohesin protein Mcd1 and the C-terminal fragment of Mcd1 induces apoptotic cell death (Figure 7; Yang et al., 2008). A similar pathway may also occur during the oxidative stress-induced apoptotic cell death, where under oxidative stress, Bir1 is degraded by the serine protease Omi/HtrA2, and Pds1 is consequently degraded (Figure 7; Walter et al., 2006). It is worth noting that other pathways may also be involved, especially in the oxidative stress-induced cell death (Mazzoni et al., 2005; Almeida et al., 2007; Lu et al., 2011).

## Conflict of Interest Statement

The authors declare that the research was conducted in the absence of any commercial or financial relationships that could be construed as a potential conflict of interest.

## Footnotes

^1^http://web.uni-frankfurt.de/fb15/mikro/euroscarf/.

^2^http://imagej.nih.gov/ij/.
